# Beyond the spore, the exosporium sugar anthrose impacts vegetative *Bacillus anthracis* gene regulation in cis and trans

**DOI:** 10.1038/s41598-023-32162-x

**Published:** 2023-03-28

**Authors:** Michael H. Norris, Andrew P. Bluhm, Morgan C. Metrailer, Treenate Jiranantasak, Alexander Kirpich, Ted Hadfield, Jose Miguel Ponciano, Jason K. Blackburn

**Affiliations:** 1 Spatial Epidemiology and Ecology Research Laboratory, Department of Geography, University of Florida, Gainesville, FL USA; 2 Emerging Pathogens Institute, University of Florida, Gainesville, FL USA; 3 Department of Population Health Sciences, School of Public Health, Georgia State University, Atlanta, GA USA; 4 Department of Biology, University of Florida, Gainesville, FL USA

**Keywords:** Genetics, Microbiology, Infectious diseases

## Abstract

The *Bacillus anthracis* exosporium nap is the outermost portion of spore that interacts with the environment and host systems. Changes to this layer have the potential to impact wide-ranging physiological and immunological processes. The unique sugar, anthrose, normally coats the exosporium nap at its most distal points. We previously identified additional mechanisms rendering *B. anthracis* anthrose negative. In this work, several new *ant* ^*−*^ *B. anthracis* strains are identified and the impact of anthrose negativity on spore physiology is investigated. We demonstrate that live-attenuated Sterne vaccines as well as culture filtrate anthrax vaccines generate antibodies targeting non-protein components of the spore. The role of anthrose as a vegetative *B. anthracis* Sterne signaling molecule is implicated by luminescent expression strain assays, RNA-seq experiments, and toxin secretion analysis by western blot. Pure anthrose and the sporulation-inducing nucleoside analogue decoyinine had similar effects on toxin expression. Co-culture experiments demonstrated gene expression changes in *B. anthracis* depend on intracellular anthrose status (cis) in addition to anthrose status of extracellular interactions (trans). These findings provide a mechanism for how a unique spore-specific sugar residue affects physiology, expression and genetics of vegetative *B. anthracis* with impacts on the ecology, pathogenesis, and vaccinology of anthrax.

## Introduction

The bacterium *Bacillus anthracis* causes anthrax and can survive harsh environmental conditions by forming a spore^1^. Surrounding the endospore is a loose protein layer, rich with carbohydrates termed the exosporium^2^. During sporulation, the exosporium is assembled around the forespore while forming in the mother cell through a coordinated effort of the CotE*,* CotO*,* and CotY proteins^3^. The outside facing portion of the exosporium is composed of glycoproteins creating a velcro-like layer known as the exosporium nap. The nap contains protruding stalks of the glycosylated BclA and BclB proteins attached to basal layer proteins ExsFA/BxpB and ExsFB^4,5^. The glycoprotein exosporium nap imparts a charged surface to the spore and is the distal surface mediating interactions between quiescent spores and the external environment, including soil particles, animal host-cells, and other spores. Upon germination, the exosporium nap is shed and *B. anthracis* begins to germinate, then replicates in vegetative form while secreting anthrax toxin^6^.

Eight proteins have been identified as significant components of the exosporium when prepared from exosporia washed to remove any vegetative cell proteins^7^. The BclA protein is the major protein component of the exosporium and forms the stalk-like nap fibers protruding from the exosporium surface. The collagen-like repeat regions of BclA vary in length between strains of *B. anthracis* depending on *bclA* gene size. These polymorphisms contribute to observable nap thickness changes on the spore surface^8^. BclA is present in trimeric formations where collagen-like regions are densely glycosylated with pentasaccharide repeats of GalNAc-Rha-Rha-Rha-Ant^9^. Ant is the monosaccharide anthrose and is a rare sugar found in few places in nature. The anthrose biosynthetic operon has been well-characterized and is composed of four genes *antA, antB, antC,* and *antD*^10,11^. All genes are involved in anthrose biosynthesis with knockout of *antA* reducing measurable spore anthrose by half and knockout of *antB*, *antC* or *antD* abolishing detectable spore anthrose levels^11^. Anthrose is not synthesized by other *Bacillus* spp. and so is uniquely present on the surface of *B. anthracis* spores. Alternative sugar residues are found on spores of other *Bacillus* spp, such as cereose present on *Bacillus cereus* spores^12,13^*.* Even though BclA is on the surface of the exosporium its contribution to pathogenesis is unclear. BclA was not required for full virulence in high dose Sterne^4^ or Ames^14^ mouse challenge experiments, while in another study a Δ*bclA* Sterne 34F2 mutant had a 50–70% reduction in LD_50_ compared to wild-type Sterne 34F2^15^. High dose study design may mask the virulence effects of *bclA* knockout with fulminant toxin and capsule production that can be revealed in more sensitive LD_50_ studies. Importantly, a BclA knockout effectively removes anthrose from the spore surface, while leaving its biosynthesis in vegetative cells intact. Knocking out BclA has been shown to increase association with epithelial cells, fibroblasts, and endothelial cells but not macrophages^16^. This was corroborated by others that showed BclA knock out spores were unable to bind to the macrophage receptor CD14 while removal of anthrose from BclA in *antC*/*degT* knock out spores increased binding to the CD14 receptor by revealing the rhamnose residues^17^. This agrees with findings that mice challenged with *bclA* mutant spores retain more spores in the bronchoalveolar lung fluid after aerosol challenge^14^. The precise function of anthrose and its contribution to pathogenesis remained unclear, with evidence supporting interaction with the soil environment and cells of the immune system. Previously, we found removing anthrose from the spore surface reduced germination efficiency and increased sporulation rates in a heterologous *B. anthracis* Sterne model^18^. Besides physiological changes, anthrose negative spores had half the LD_50_ in a subcutaneous mouse challenge model leading to a more rapid time to death and faster dissemination in host organs. Increase in lethality was also observed in a second animal model by challenging *Galleria mellonella* larvae with spores^18^.

Lack of anthrose was previously thought to be limited to a sub-group of unique *B. anthracis* isolated in Chad, Mali, Cameroon^19^ and Nigeria aptly dubbed the West Africa Group (WAG)^20^. These strains have a conserved SNP and nucleotide triplication event that renders them *ant* ^*−*^. We previously identified two strains of *B. anthracis* genetically *ant—* via chromosomal deletions encompassing the entirety of the anthrose biosynthetic operon, one from Chile and another from Poland, in our *B. anthracis* global collection^18,21^. A search of publicly available sequence records indicated *B. anthracis* strain Ba4599 Heroin, which was isolated from a European anthrax case linked to *B. anthracis* spore-contaminated heroin, had a novel SNP linked to the *ant* ^*−*^ genotype. These three observations expanded the mechanisms and geographic distribution of anthrose negative strains beyond the original WAG observations placing more urgency on understanding their geographic origins and implications of spore anthrose loss.

Here we analyze anthrose negativity beginning from an epidemiological perspective as we understand the breadth of accumulating anthrose mutations. By examining recently deposited next generation sequencing files we expand our knowledge of the geographic distribution of anthrose negative strains and associate them with outbreaks of public health importance. This provides the context for analyzing the role anthrose may play in the cell and how *B. anthracis* is affected by its absence. Understanding the consequences of anthrose loss on physical properties of the spore would indicate whether anthrose imbues innate properties to the exosporium nap. Besides physical properties of the spore surface, many bacteria respond to biosynthetic metabolites, like lactose or arabinose, and modify gene expression accordingly. Many previous studies removed BclA from the surface of the spore by gene mutation, this would not impact the production of anthrose in the cell towards sporulation. To dissect the role of anthrose in bacterial cell physiology we focused on comparing wild-type and anthrose mutants in this work. We sought to characterize global gene expression shifts in response to the unique spore decorating monosaccharide, anthrose. Anthrose could cause shifts in the *B. anthracis* transcriptome during vegetative growth by serving as genetic inducer/or repressor as part of the metabolic flux occurring along the path to sporulation. This would serve as an active selective pressure for mutation of the anthrose operon during vegetative growth. More specifically, important virulence mechanisms associated with vegetative growth, such as toxin secretion, may be affected by anthrose flux. RNA-seq in association with luminescent reporter strain experiments were used to probe gene expression in the presence of anthrose. A closer look at the changed immunological properties of the spore provides more evidence that spore surface epitope modification may evade associated immune responses. We produced several luminescent reporter strains in the *B. anthracis* Sterne background to characterize the effect of anthrose status on gene induction over time. Treatment of *B. anthracis* luminescent reporters with purified anthrose and decoyinine (a *Bacillus* sporulation inducing GMP synthatase inhibitor produced by *Streptomyces*) revealed regulatory differences in anthrose positive and negative strains. Finally, co-culture of luminescent reporters with anthrose positive and negative strains was used to investigate if native anthrose levels changed gene expression in neighboring cells. Taken together, this work frames anthrose negativity as a phenotypic mutation that can impact physiology of vegetative *B. anthracis* while changing the structure of the spore.

## Results

### Summary of known *ant*^*-*^ strains and new mutations found

**Figure 1:**
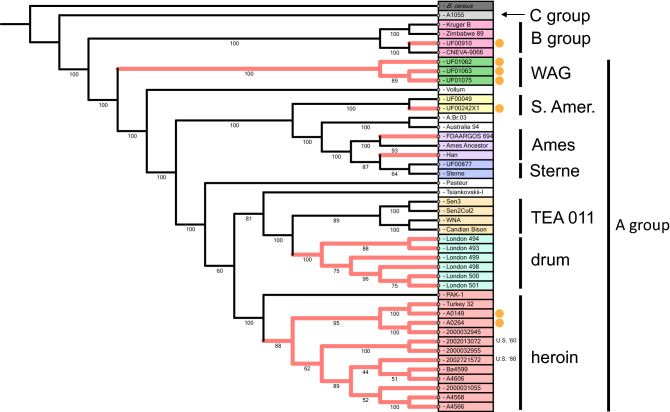
Whole genome SNP tree of known *ant* ^*-*^ *Bacillus anthracis* strains in relation to other *B. anthracis* lineages. Various *B. anthracis* groups and lineages of interest to this work are labeled on the right side of the figure. The orange dots indicate strains in our collection that are anthrose negative. The different colored labels are to help differentiate the lineages. The red highlighted branch labels indicate *B. anthracis* strains that are anthrose negative by whole genome sequence interrogation. *B. cereus* is natively anthrose negative, not because of discreet mutations, and so the branch is not highlighted red. The branch lengths are indicated above the branches with bootstrap values out of 100 located underneath. The tree has been rooted to the *B. cereus* outgroup. Diverse strains across lineages are acquiring anthrose negativity in diverse and independent ways. The whole genome SNP phylogenetic tree in this figure was produced by analysis with PhaME^54^ and visualized with iTOL^55^.

A review of our previous work and in-depth analysis reveals several genetic mechanisms for anthrose loss. Coupled with these diverse mechanisms, the geographic distribution of *ant* ^*-*^ strains is wide (across at least 15 countries) and long-spanning (more than 70 years). Analysis of the known *ant* ^*-*^ strains in relation to several type strains and other *B. anthracis* strains of interest by whole genome SNP comparison shows their relatedness to other *B. anthracis* type strains and each other (Fig. 1). Label colors represent different *B. anthracis* lineages and lineages important to this work. The outgroup *Bacillus cereus* grouped closest to the C group representative strain A1055. A summary of *ant* ^*-*^ genetic signatures, strains, and relevant epidemiological data are present in Table 1.

**Table 1:** Summary of identified *ant* ^*-*^ mutations, the strains they are found in, geographic source, and date range.

*ant*^-^ mutation	Strain	Isolate location (phylogeographic source)	Year
*antB* SNP C892T (stop)	UF01063, UF01075 (WAG strains)	Nigeria, Chad, Mali, Cameroon	1950s
*antB* ∆1013 (frameshift)	Han	Liaoning, China	2012
*antB* ∆1473 (frameshift)	FDAARGOS_694, London_493, London_494, London_498, London_499, London_500, London_501	UK (Senegal/Gambia)	2006
*antB* SNP G418T (stop)	Ba4599 Heroin, UF00026, UF00430, A4566, A4568, A4606, 2000032945, 2000032955, 2002013072, 2002721572, 2000031055	Denmark, Norway, Scotland, United States, Turkey (heroin clade)	1957–2012
*antC* AAAAAAAG trip (stop)	UF01063, UF01075 (WAG strains)	Nigeria, Chad, Mali, Cameroon	1950s
19 kbp deletion surrounding anthrose operon	UF00242X1	Chile	2001
59 kbp deletion surrounding anthrose operon	UF00910	Poland	1999

### Electron microscopy and analysis of the effects of anthrose removal on the *B. anthracis* exosporium nap

**Figure 2:**
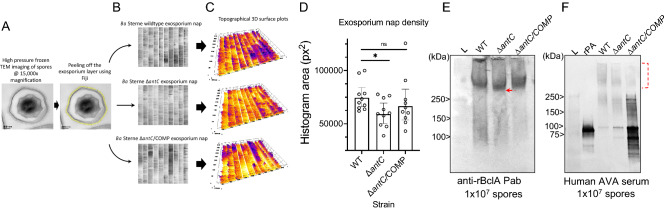
Analysis of the exosporium by fiber topographical surface plot and western blot. (**A**) An example of a single spore image that was analyzed using Fiji to ‘peel’ the exosporium nap layer from the spore surface. (**B**) Ten randomly selected *B. anthracis* Sterne WT, Δ*antC*, Δ*antC*/COMP spores had their exosporium nap arrayed. (**C**) These same ten spores had their nap layers converted to a topographical 3D surface plot to ease visualization of the electron density associated with the TEM images. (**D**) The black and white images in (**B**) were converted to histograms and their areas determined. *p < 0.05; ns = not significant. Individual values, averages and 95% CI are shown. (**E**) Polyclonal Ab to rBclA was used to blot 1 × 10^7^ purified spores of each strain. The red arrow indicates a slight decrease in molecular weight associated with deletion of anthrose. (**F**) The same spore preps in (**E**) were blotted against pooled human AVA serum, indicating immune reactivity to spore antigen. High molecular weight regions coinciding with BclA reactivity in (**E**) are indicated by the dashed bracket.

To observe physical alteration of the exosporium nap occurring in the absence of anthrose, transmission electron microscopy of the spores was carried out. Spores prepared from *B. anthracis* Sterne WT, *B. anthracis* Sterne Δ*antC,* and *B. anthracis* Sterne Δ*antC/*COMP were fixed and submitted to high-pressure freezing then observed at 15,000 × magnification (Fig . S1A–F). The exosporium nap of ten spore images from two different preparations were digitally peeled to linearize and visualize a 3D topographical heat map (Fig. 2A–C). Qualitatively the images show the nap of the WT strain is more electron dense (more purple) compared to the anthrose negative Δ*antC* mutant (Fig. 2C). The anthrose complement spores have very dense regions of nap which were generally more irregular in density across the whole spore. Histograms as a measure of pixel area were generated from the linearized nap images and used to quantitively compare the exosporium fiber density between strains (Fig. 2D). The data showed the density of the exosporium nap fibers was the lowest in the Δ*antC* mutant while the WT and Δ*antC/* COMP had similar densities.

### Reactivity of immune plasma and serum to vegetative and spore components

Western blots were performed on an equal number of spores from the WT, Δ*antC,* or Δ*antC/* COMP strain to assess if anthrose loss affected apparent BclA size and reactivity with immune serum. Removal of anthrose could expose BclA epitopes that are otherwise masked by hydrophobic sugar moieties, with implications on the immune repertoire associated with anthrax vaccines. Polyclonal antibody to recombinant BclA protein, the protein decorated with the anthrose-tipped pentasaccharide, was used to detect BclA protein size (Fig. 2E). BclA is a ~ 21 kDa protein that can run at > 150 kDa on an SDS-PAGE gel because of its numerous polysaccharide modifications. A downward shift is evident when blotting spores lacking anthrose (Δ*antC*). Blotting of the same spore preparations with pooled anthrax vaccine adsorbed (AVA)-vaccinated human serum show the human serum has moderately less binding to Δ*antC* spores compared to WT in the high-molecular weight region of BclA region while having increased binding in the lower molecular weight BclA and PA region (Fig. 2F). To further investigate the reactivity of vaccine serum to non-protein bacterial components in vegetative bacteria and spores, protein was degraded with proteinase-K then blotted with rabbit anti-*B. anthracis* polyclonal antibody, pooled human AVA plasma, Sterne-vaccinated bison serum, and naïve bison serum (Fig. S2A–E). Naïve bison serum was unreactive to all samples run on the gel (Fig. S2D). The immune serum samples reacted strongly with untreated vegetative cells (lanes 1) coinciding to a protein migrating at ~ 83 kDa; the same as PA (Fig. S2B–D). Vegetative cell lysates treated with proteinase-K to degrade proteins (lane 2) showed little reactivity with the immune serums. Lane 3 of each blot are spore lysates. Immune samples appear to react with PA from spore lysates. PA can bind to the outside of spores^22^. High molecular weight bands specific to spores are present. When the proteins are degraded by proteinase K treatment, a high molecular weight material continues to react with each immune sample. This high molecular weight material that is proteinase-K resistant coincides with heavily glycosylated BclA protein specific to the spore. The Sterne vaccine is a live attenuated spore vaccine, so it is not surprising the bison serum sample reacted strongly to spore specific non-protein antigen (Fig. S2D). The AVA vaccine is produced from precipitated culture filtrate from a vegetative non-encapsulated *B. anthracis* Sterne strain (as is the anthrax vaccine precipitated (AVP) vaccine); similar strains are the live-attenuated spores used in the veterinary vaccine^23^. The *B. anthracis* strain used for AVA production is V770-NP1-R^24^. This strain is grown anaerobically in a fermenter and culture filtrate is adsorbed to alhydrogel. *B. anthracis* V770-NP1-R is a non-proteolytic pXO2-negative derivative of strain V770^1^ that was isolated from a bovine anthrax case in Florida in 1951^25^. The blots show reactivity to non-protein spore-specific material, indicating a small amount of spore specific antigen is present in AVA (Fig. S2E). An analysis of the similarly produced AVP vaccine from the UK did observe spores in vaccine production vessels, however the investigators concluded with a dearth of supporting data that this was due to failure of 30% of the inoculum to germinate^26^. Proteomic analyses of the AVP vaccine found the major components to be PA (64%), LF (8%), and EF (3%) and 258 other proteins making up the other 25%, non-protein components were not analyzed^27^. BclA is the immunodominant protein on the spore and its change or modification, such as anthrose removal, could modify immunoreactivity in human and animal hosts.

During growth of the mutant, increased clumping of cells in shaking broth cultures was observed. Microscopic analysis of cells over time showed the mutant formed longer chains of vegetative cells generating biofilm like structures as the bacteria sporulated (Fig. S3), indicating a potential global role for anthrose sensing in the physiology of *B. anthracis.* The mutants were whole genome sequenced and no mutations of consequence were detected to explain the observed behavior of our *antC* mutant.

**Figure 3:**
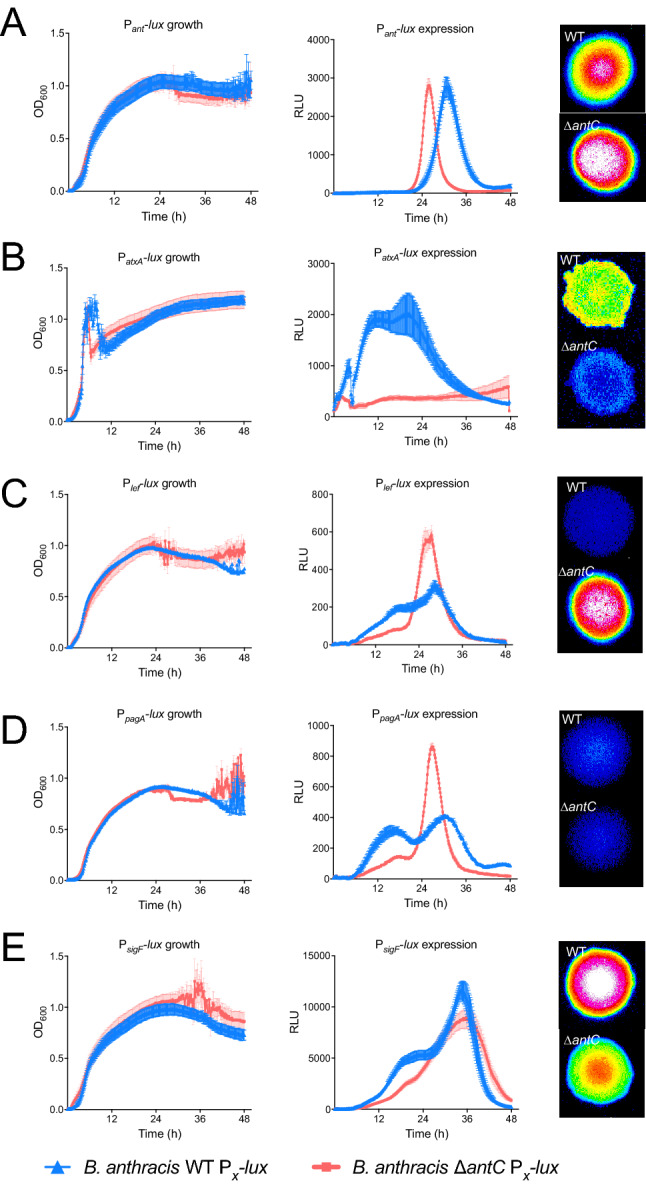
Luminescent expression patterns from important *B. anthracis* promoters are affected by anthrose status. Growth and luminescent expression experiments in HIB + Km10 were used to characterize expression of *lux* from the (**A**) P_*ant*_, (**B**) P_*atxA*_, (**C**) P_*lef*_, (**D**) P_*pagA*_ and (**E**) P_*sigF*_ promoters over 48 h. In each graph, growth (OD at 600 nm; first column of graphs) or luminescence (RLU; second column of graphs) of the Sterne WT is in blue and the Sterne Δ*antC* mutants is in red. Luminescent imaging of solid plate colonies at 24 h are below the broth time courses. Growth and luminescent curve data from two independent experiments carried out in triplicate with the mean and standard error of the mean at each timepoint shown.

### Gene expression and toxin secretion levels in *B. anthracis* Sterne are perturbed by knocking out anthrose biosynthesis

Five luminescent reporter plasmids were generated to allow expression analysis from the anthrose operon promoter (P_*ant*_) the *atxA* promoter (P_*atxA*_), the lethal factor promoter (P_*lef*_), the *pagA* promoter (P_*pagA*_), and the *sigF* promoter (P_*sigF*_ aka the *spoIIAA*-*spoIIAB*-*sigF* promoter). These plasmids were conjugated into *B.anthracis* Sterne and *B.anthracis* Sterne Δ*antC* that we previously verified were unable to produce anthrose ^18^. The resulting strains were grown in triplicate in Heart Infusion Broth (HIB) a high protein content medium devoid of sugars and spotted on solid HIB agar. RLU were measured every 10 min for 48 h in broth and imaged at 24 h on solid media (Fig. 3A–E). Knocking out anthrose production shifts peak P_*ant*_ expression from 32 to 26 h indicating presence of anthrose can repress expression of its own biosynthetic operon (Fig. 3A). Expression from P_*atxA*_ is greatly reduced in the anthrose mutant (Fig. 3B). While expression of P_*lef*_ and P_*pagA*_ were initially delayed in the anthrose mutant compared to WT, large spikes at 24 h indicate increased levels of these toxin components as the mutant enters stationary phase (red lines in Fig. 3C and D). Since anthrose is present on the exosporium of the mature spore, the first forespore specific sigma factor, *sigF*, reporter was used to measure if knockout of anthrose production affected this dedicated step. Expression from P_*sigF*_ increased at a slower rate in the anthrose mutant, then eventually approached the levels seen in the Sterne WT (Fig. 3E).

**Figure 4:**
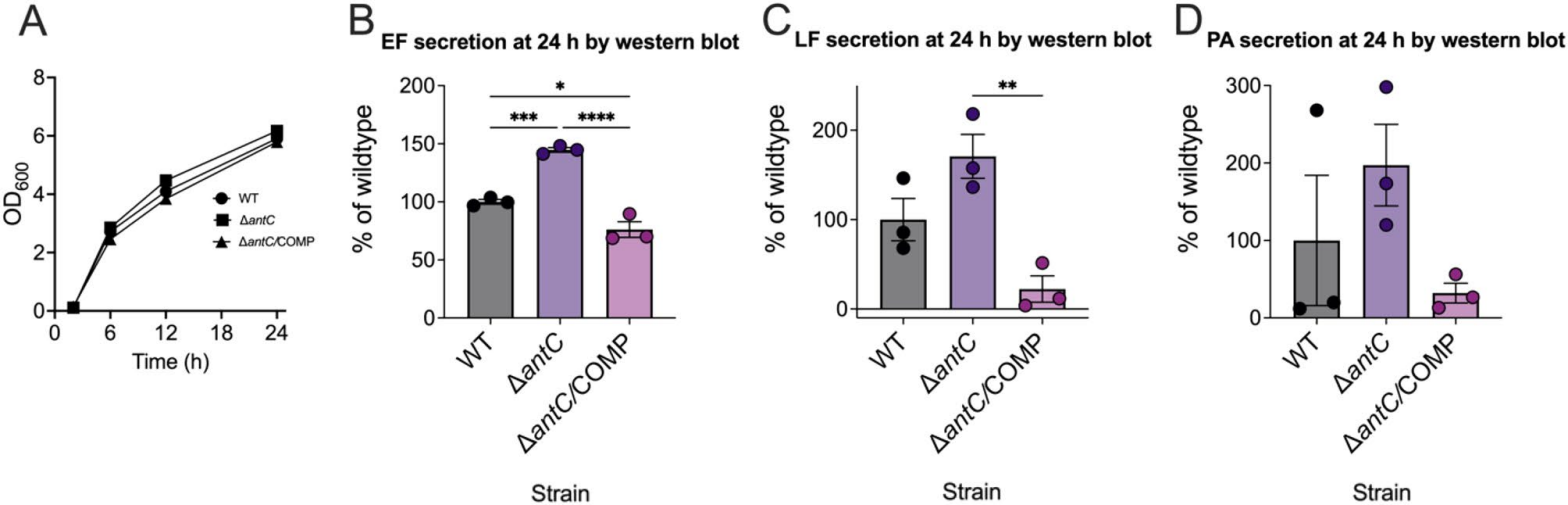
Edema factor, lethal factor and protective antigen secretion by *B. anthracis* Sterne 34F2 are perturbed in the absence of anthrose. To confirm luminescent expression studies *B. anthracis* Sterne WT, Δ*antC*, and Δ*antC*/COMP strains were grown in HIB + Km 10 in the presence of protease inhibitors in triplicate, filter purified 24 h supernatant samples. The ODs at 600 nm were measured over time (**A**) and the supernatants were blotted for (**B**) Edema factor, (**C**) Lethal factor, or (**D**) Protective antigen. Individual band intensities, their average, and SD are shown as a percent of wildtype (WT). A repeated measures one-way ANOVA was run on the growth curve timepoints and the optical densities were not significantly different than WT in (**A**). One-way ANOVA tests were used to determine significant differences of the band intensities in (**B**–**D**). *p < 0.05, **p < 0.01, ***p < 0.001, ****p < 0.0001.

To verify anthrose involvement in toxin secretion, triplicate cultures of WT Sterne, Δ*antC*, and Δ*antC/*COMP were grown in 200 ml culture flasks of HIB + protease inhibitors and supernatants were collected at 24 h (Fig. 4A). Filtered supernatants were analyzed in triplicate by western blot for EF, LF, and PA. These findings indicated significantly increased levels of EF in the Δ*antC* supernatant compared to WT and the complement (Fig. 4B). LF levels in Δ*antC* supernatant were elevated, although not significantly, compared to WT. The Δ*antC* levels compared to the Δ*antC/*COMP were significantly different (Fig. 4C). The trend observed with LF was observed in PA blots, however high variability led to statistically insignificant differences (Fig. 4D). These data indicate all supernatant toxin levels were perturbed by the Δ*antC* mutation in Sterne; some significantly.

### Anthrose as a global transcriptional modulator in vegetative cells

**Figure 5:**
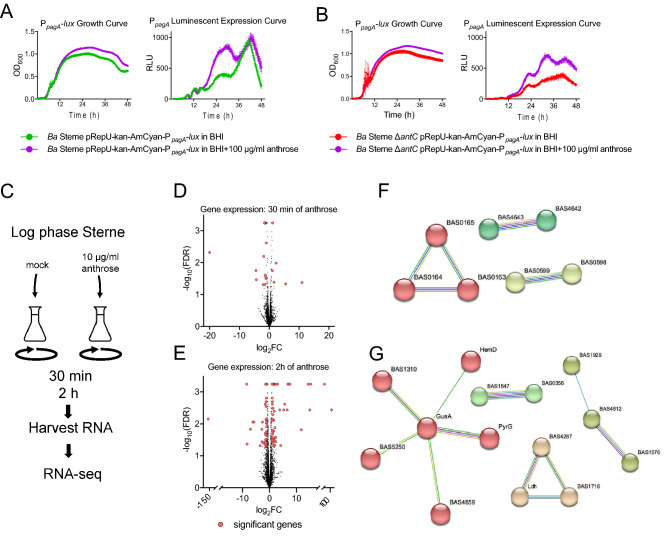
Anthrose induces changes in expression of protective antigen and affects global gene regulation in *B. anthracis* Sterne. (**A**) The *B. anthracis* Sterne protective antigen (PA) luminescent reporter strain was grown with (purple line) and without (green line) 100 μg/ml pure anthrose for 48 h. (**B**) The *B. anthracis* Sterne Δ*antC* protective antigen (PA) luminescent reporter strain was grown in BHI broth with (purple line) and without (red line)100 μg/ml pure anthrose for 48 h. Growth curves were carried out in triplicate with the OD at 600 nm and the average relative luminescent units (RLU) and standard error of the mean presented at each time point. (**C**) Experimental design to measure global transcriptomic levels at 30 min and 2 h after adding 10 μg/ml of pure anthrose to log-phase *B. anthracis* Sterne grown in BHI. Each sample was collected from three experiments and submitted to RNA-seq. (**D**) Gene expression of *B. anthracis* Sterne 30 min after addition of 10 μg/ml of pure anthrose compared to mock (water) treated culture grown in parallel and (**E**) after 2 h compared to mock (water) treated culture grown in parallel. Red dots indicate significant genes that experience a fold-change greater than 2 or less than -2 and have a false discovery rate less than 0.05. (**F**) String network functional analysis of 30 min gene clusters with BAS loci labels. The red cluster are related to glucokinase processes, the teal are ATP-transmembrane processes, and yellow are involved in sugar metabolism. Lines connecting genes are different evidence of interaction. (**G**) String network functional analysis of 2 h gene clusters with BAS locus labels. Nucleoside monophosphate (GMP and CTP) biosynthetic processes are part of the red cluster, the salmon group is glycolytic processes, yellow are other carbon metabolic processes, and green are involved in chemotaxis/two-component systems. Lines connecting genes are different evidence of interaction.

In preliminary experiments, the absence of anthrose in our genetic Sterne knockout was found to decrease *pagA* expression in BHI broth containing glucose (Fig. 5A and B). This contrasted with the large spike in *pagA* expression when the Sterne Δ*antC* mutant is grown in protein rich HIB medium a which does not contain sugars. When exogenous anthrose was added during growth in BHI broth, expression from P_*pagA*_ increased in both WT (purple line in Fig. 5A) and Δ*antC* Sterne (purple line in Fig. 5B). Many bacteria respond to levels of metabolic intermediates and modulate gene expression accordingly. We hypothesized intracellular or extracellular anthrose levels could impact the vegetative cells through modification of gene expression during vegetative growth. Wildtype *B. anthracis* Sterne was grown in the presence and absence of exogenously added pure anthrose to avoid any possibility of confounding data in a deletion mutant. Bacteria were harvested at two timepoints post-addition of anthrose, 30 min and 2 h, and compared to diluent only treated cultures (Fig. 5C). After 30 min treatment, there were 6 genes significantly upregulated more than two-fold while 17 genes were downregulated (Fig. 5D). These genes were mainly involved in generating metabolic intermediates such as pyruvate and trehalose. The most upregulated gene was a putative membrane protein (log_2_FC = 10.98) followed by a gene encoding a GerPF homologue at a log_2_FC of 10.98. Mutation of GerPF family proteins have been linked to a super-dormant spore phenotype^28^. The gene experiencing the highest downregulation was a putative lipoprotein (log_2_FC = − 20.02). No genes located on pXO1 were detected at 30 min. Differentially transcribed genes from the 30 min timepoint are listed in Table 2. After 2 h of anthrose treatment, 52 genes experienced significant up regulation, 18 of which were hypothetical proteins (Fig. 5E). Forty-five genes experienced significant down regulation across replicates, 13 of which were hypothetical proteins. A YfhD-like sporulation-specific sigma-F regulated protein experienced a log_2_FC of 116.33. YfhD-family proteins are transglycosylases involved in peptidoglycan degradation. This gene is expressed in the *Bacillus subtilis* prespore^29^ and is one of the most abundant mRNAs in dormant *B. subtilis* spores^30^. Expression of this protein is also regulated by a sporulation specific sigma factor F. Three hypothetical genes from pXO1 were upregulated (log_2_FC of AW20_5643 = 32.20, AW20_5714 = 14.91, and AW20_5607 = 5.66). AW20_5643 (log_2_FC of 32.20) encodes a nuclease-domain containing protein and is immediately downstream from *lef.* Three genes, all hypothetical, from pXO1 were significantly downregulated greater than two-fold: AW20_5645 (log_2_FC = − 7.07), AW20_5667 (log_2_FC = − 1.45), and AW20_5770 (log_2_FC = − 1.16). AW20_5667 is immediately upstream and divergently transcribed from *cya* while AW20_5645 is between *lef* and *pagR*. The *cya, lef,* and *pag* genes expression changes were not statistically significant however the transcriptional activator *atxA* (AW20_5658) did experience statistically significant repression of log_2_FC = − 0.62. Additionally, there were transcriptional regulators and other spore-specific genes identified at the 2 h timepoint. Differentially transcribed genes from the 2 h timepoint, including the genes found on pXO1 are listed in Table 3. These data indicate anthrose plays a role in the transcriptional landscape of vegetatively growing *B. anthracis* by serving as an inducer/repressor molecule of a yet uncharacterized regulon. STRING network analysis was used to identify functional clusters of genes in the 30 min (Fig. 5F) and 2 h data sets (Fig. 5G). Interestingly a cluster of GMP synthetase genes was identified in the 2 h dataset. If anthrose acts as a GMP synthetase inhibitor (to reduce GTP levels, as the evidence suggests), the bacteria may up regulate *guaA* to overcompensate. A two-component chemotaxis system was also identified.

**Table 2:** Significant genes from the 30 min timepoint. Gene loci are listed according to NCBI Sterne RefSeq assembly GCF_000832635.1 (Locus AW20) with the RefSeq assembly GCF_000008165.1 loci provided (Locus BAS) where available. Protein name and functions are according to PATRIC and the log_2_FC are shown.

Locus (AW20)	Locus (BAS)	Protein id	log_2_FC
AW20_1638	BAS1044	Putative membrane protein	10.98
AW20_3494	BAS4692	GerPF/GerPA protein family	5.51
AW20_3546	BAS4643	LolD; ABC transporter-like sensor ATP-binding protein	2.30
AW20_398	BAS2257	CspA; cold shock protein	1.63
AW20_831	BAS1830	Cell envelope-associated transcriptional attenuator LytR-CpsA-Psr	1.17
AW20_3547	BAS4642	ABC-type antimicrobial peptide transport system, permease component	1.07
AW20_3285	BAS4875	Long chain acyl-CoA dehydrogenase (fadN-fadA-fadE operon)	− 1.00
AW20_905	BAS1756	Pdp; Pyrimidine-nucleoside phosphorylase	− 1.09
AW20_2627	BAS0163	FGGY family of carbohydrate kinase	− 1.11
AW20_2257	BAS0482	PflA; Pyruvate formate-lyase activating enzyme	− 1.18
AW20_2081	BAS0631	Ribose operon repressor; transcriptional regulator LacI/PurR family	− 1.20
AW20_2116	BAS0598	TreB; PTS system, trehalose-specific IIBC component	− 1.26
AW20_5282	BAS2949	Magnesium and cobalt transport protein CorA	− 1.33
AW20_3236	BAS4922	Nucleoside transporter, NupC family	− 1.45
AW20_2625	BAS0165	GntP; Gluconate permease, Bsu4004 homolog	− 1.45
AW20_2115	BAS0599	TreC; Trehalose-6-phosphate hydrolase	− 1.46
AW20_453	BAS2203	Oxalate/formate antiporter	− 1.57
AW20_2626	BAS0164	FGGY family of carbohydrate kinase; gluconokinase	− 1.60
AW20_2866	BAS5293	LrgA-associated membrane protein LrgB	− 1.87
AW20_2865	BAS5294	Antiholin-like protein LrgA	− 2.05
AW20_376		Hypothetical protein	− 4.34
AW20_4692	BAS3529	Hypothetical protein; PDDEXK_nuclease-like	− 4.47
AW20_2596	BAS0193	Putative lipoprotein	− 20.02

**Table 3:** Significant genes from the the 2 h timepoint. *atxA* did not experience a significant fold-change however the FDR did make the cutoff. Bolded entries indicate the gene is present on the virulence plasmid pXO1. Gene loci are listed according to NCBI Sterne RefSeq assembly GCF_000832635.1 (Locus AW20) with the RefSeq assembly GCF_000008165.1 loci provided (Locus BAS) where available. Protein name and functions are according to PATRIC and the log_2_FC are shown.

Locus (AW20)	Locus (BAS)	Protein id	log_2_FC
AW20_4874	BAS3350	Hypothetical protein; YfhD-like sporulation-specific sigma-F regulated	116.33
**AW20_5643**		**Hypothetical protein; nuclease domain protein downstream of *lef,* pXO1**	**32.20**
AW20_1990		Hypothetical protein	15.27
**AW20_5714**		**Hypothetical protein, pXO1**	14.91
AW20_21		Acetyltransferase, GNAT family	14.33
AW20_3821	BAS4374	FIG016877: hypothetical protein	11.80
AW20_4418	BAS3801	Prophage pi2 protein 35	7.59
AW20_3318	BAS4843	Uncharacterized DUF1805-containing protein YunC	7.23
AW20_705	BAS1952	Hypothetical protein	6.84
AW20_4729	BAS3492	Hypothetical protein; prophage LambdaBa01, transcriptional regulator, AbrB family	6.56
**AW20_5607**		**Hypothetical protein, pXO1**	**5.66**
AW20_1773	BAS0913	Hypothetical protein	5.32
AW20_286	BAS2363	Hypothetical protein, GBAA2540 homolog	4.18
AW20_4403	BAS3814	Hypothetical protein	3.79
AW20_1378	BAS1298	Hypothetical protein, regulatory YrvL family protein	3.00
AW20_3164	BAS4995	Stage V sporulation protein AC (SpoVAC)	2.80
AW20_2179	BAS0537	Glycerol-3-phosphate ABC transporter, permease protein UgpE	2.35
AW20_1941	BAS0749	Uncharacterized MFS-type transporter	2.26
AW20_1364	BAS1310	Ketol-acid reductoisomerase (NADP( +))	2.11
AW20_981	BAS1681	MutT/Nudix family protein	2.00
AW20_170	BAS2472	EDD, DegV family protein	1.93
AW20_4951	BAS3273	Hypothetical protein	1.88
AW20_2438	BAS0315	Polysaccharide deacetylase	1.82
AW20_917	BAS1745	Hypothetical protein	1.74
**AW20_5718**		**Hypothetical protein, pXO1**	**1.70**
AW20_3836	BAS4360	Uroporphyrinogen-III synthase	1.62
AW20_5499	BAS2739	Hypothetical protein	1.60
AW20_5054	BAS3171	ATP synthase protein I	1.59
AW20_163	BAS2480	Hypothetical protein	1.58
AW20_2395	BAS0356	Methyl-accepting chemotaxis protein	1.52
AW20_4704	BAS3516	Hypothetical protein	1.47
AW20_330	BAS2320	Hypothetical protein	1.46
AW20_2243	BAS0493	Small acid-soluble spore protein, gamma-type SASP	1.38
AW20_3302	BAS4859	Glycine cleavage system H protein	1.38
AW20_720	BAS1937	Uncharacterized membrane protein Bsu0528 (YdeO)	1.29
AW20_4754	BAS3468	UPF0154 protein YneF	1.24
AW20_1933	BAS0757	Enterotoxin / cell-wall binding protein	1.23
AW20_1605	BAS1076	Catalase KatE	1.22
AW20_944	BAS1718	Threonine dehydratase biosynthetic	1.21
AW20_1190	BAS1482	Uncharacterized protein YpbS	1.21
AW20_594	BAS2061	Hydrolase, alpha/beta fold family	1.20
AW20_2908	BAS5250	Uracil-DNA glycosylase, family 1	1.18
AW20_1379	BAS1297	Uncharacterized protein SAV1929	1.13
AW20_2124	BAS0590	Nitric oxide reductase activation protein NorD	1.09
AW20_2878	BAS5280	Glycosyl transferase, group 2 family	1.07
AW20_2879	BAS5279	Membrane protein exporting O-antigen, teichoic acid lipoteichoic acids	1.05
AW20_3899	BAS4296	HesA/MoeB/ThiF family protein	1.04
AW20_877	BAS1784	L-lactate dehydrogenase	1.04
AW20_4199	BAS4003	Acetyltransferase, GNAT family	1.02
AW20_2510	BAS0254	GMP synthase [glutamine-hydrolyzing], amidotransferase subunit	1.02
AW20_2971	BAS5187	CTP synthase	1.01
AW20_918	BAS1744	Rhodanese domain protein UPF0176, Firmicutes subgroup	1.00
AW20_1906	BAS0785	Outer surface protein of unknown function, cellobiose operon	− 1.00
AW20_5111	BAS3116	Uncharacterized membrane protein YwiC	− 1.01
AW20_4184		Hypothetical protein	− 1.03
AW20_983	BAS1679	Uncharacterized protein YndH	− 1.06
AW20_5555	BAS2692	Integral membrane protein; YeaQ/YmgE family	− 1.06
AW20_728	BAS1929	Phosphoglycerate mutase family 3	− 1.06
AW20_1125	BAS1547	Chemotaxis protein methyltransferase CheR	− 1.11
AW20_1033	BAS1630	Transcriptional regulator, GntR family	− 1.12
AW20_4348	BAS3861	UPF0358 protein YlaN	− 1.12
AW20_4085	BAS4114	Transcriptional regulator, AcrR family	− 1.13
**AW20_5770**		**Hypothetical protein, pXO1**	− **1.16**
AW20_4157	BAS4044	Probable metallo-hydrolase YqjP	− 1.17
AW20_3880	BAS4316	Holliday junction ATP-dependent DNA helicase RuvA	− 1.18
AW20_408	BAS2248	Acetyltransferase, GNAT family	− 1.19
AW20_1384	BAS1293	Transcriptional regulator, Bla/Mec family	− 1.19
AW20_2259	BAS0480	Glycosyl transferase, group 2 family	− 1.19
AW20_2489	BAS0269	Undecaprenyl-diphosphatase (EC 3.6.1.27)	− 1.19
AW20_1586	BAS1095	3-oxoacyl-[acyl-carrier-protein] synthase, KASIII (EC 2.3.1.180)	− 1.20
AW20_3574	BAS4612	Glucose 1-dehydrogenase (EC 1.1.1.47)	− 1.21
AW20_125	BAS2516	GNAT family acetyltransferase BA2701	− 1.21
AW20_3613	BAS4574	Hypothetical protein	− 1.22
AW20_4494	BAS3724	UPF0701 protein YicC	− 1.23
AW20_4337	BAS3872	DUF1054 superfamily protein	− 1.23
AW20_5265	BAS2966	Manganese ABC transporter, ATP-binding protein SitB	− 1.27
AW20_4091	BAS4108	Mn-dependent transcriptional regulator MntR	− 1.29
AW20_5476	BAS2761	Hypothetical protein	− 1.43
AW20_3618	BAS4568	Virulence factor MviM	− 1.44
AW20_1154	BAS1518	Putative cation/acetate symporter	− 1.44
**AW20_5667**		**Hypothetical protein; cytolysin domain upstream from *cya,* pXO1**	− **1.45**
AW20_3928	BAS4267	Acetaldehyde/alcohol dehydrogenase	− 1.46
AW20_3102	BAS5058	PTS system, cellobiose-specific IIC component	− 1.49
AW20_1735	BAS0950	Putative ring-cleaving dioxygenase MhqA	− 1.51
AW20_4872	BAS3352	Uncharacterized membrane protein YuiD	-1.60
AW20_1801	BAS0887	Transcriptional regulator, TetR family	− 1.67
AW20_4647	BAS3570	Gamma-aminobutyrate (GABA) permease	− 2.38
AW20_4254	BAS3950	Hypothetical protein	− 2.59
AW20_5232	BAS2997	Hypothetical protein	− 2.75
AW20_2269	BAS0470	Hypothetical protein	− 2.85
AW20_360	BAS2292	Hypothetical protein	− 3.47
AW20_817	BAS1844	Small acid-soluble spore protein, alpha/beta family, SASP_1	− 5.79
AW20_1924	BAS0767	Polypeptide composition of the spore coat protein CotJB	− 6.18
**AW20_5645**		**Hypothetical protein DUF4037, between *lef* and *pagR,* pXO1**	− **7.07**
AW20_1760	BAS0925	Hypothetical protein	− 8.30
AW20_5242		Hypothetical protein	− 8.42
AW20_4375		Hypothetical protein	− 151.92
**AW20_5658**		**Transcriptional activator AtxA, (pXO1-119)**	− **0.62**

### Secretion of toxin components by Sterne indicates anthrose dependency

**Figure 6:**
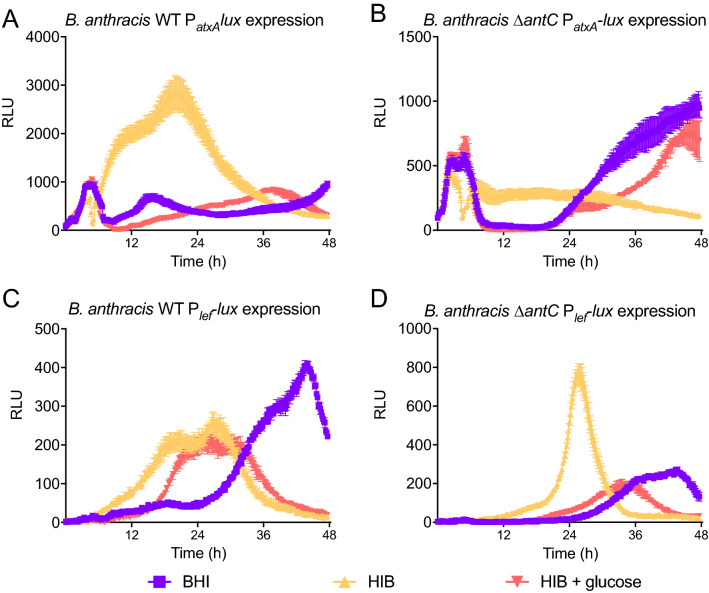
Luminescent expression patterns of *B. anthracis* virulence related promoters are affected by nutrient components. Luminescent expression patterns of (**A**) Sterne WT P_*atxA*_-*lux* (**B**) Sterne Δ*antC* P_*atxA*_-*lux* (**C**) Sterne WT P_*lef*_-*lux* and (**D**) Sterne Δ*antC* P_*lef*_-*lux*, (grown in BHI + Km 10 (purple lines), HIB + Km10 (orange lines), or HIB + Km10 + 2 mg/ml glucose (red lines) show anthrose status has different effects on expression in high protein versus high sugar nutrient conditions.

Our observation that pure anthrose (itself a sugar) decreased *pagA* expression and globally perturbs genes led us to investigate whether anthrose presence is involved in virulence expression modification through carbohydrate metabolism. With 2 mg/ml glucose, BHI broth contains high levels of sugar in addition to rich protein sources. Heart infusion broth (HIB) is essentially the same medium as BHI without dextrose. Luminescent promoter fusions for *lef* and *atxA* were introduced into Sterne WT and the Sterne Δ*antC* mutant and grown in BHI, HIB, and HIB + 2 mg/ml glucose to measure their expression in high protein and high protein + carbohydrate media (Fig. 6A–D). What can immediately be appreciated throughout the figure panels is the expression trends in BHI (purple lines) and HIB + 2 mg/ml glucose (red lines) tend to be more like each other than to expression in HIB (orange). Expression patterns from P_*lef*_ in BHI (purple lines in Fig. 4A and B) are similar to those observed for P_*pagA*_ in Fig. 5A and B. Expression from P_*atxA*_ in WT Sterne shows a prominent initial peak in a medium containing glucose then decreases and is variable thereafter (Fig. 6A purple and red lines). In HIB medium, WT P_*atxA*_ expression shows a high level of induction with RLU three times higher than in BHI or HIB + glucose (Fig. 6A orange lines). P_*atxA*_ expression in Δ*antC* Sterne grown in BHI and HIB + 2 mg/ml glucose shows the initial spike in expression then flatlines until the bacteria enter stationary phase where expression increases again around 24 h (Fig. 6B purple and red line). P_*atxA*_ expression in Δ*antC* Sterne grown in HIB is lower compared to the WT Sterne, indicating the production of anthrose by bacteria in HIB may affect *atxA* expression. In HIB, expression from P_*lef*_ in the WT occurs in a biphasic peak with a maximum 250 RLU (Fig. 6C orange line). In Fig. 6D, expression from P_*lef*_ in Δ*antC* Sterne grown in HIB is detectable as a prominent single peak at 24 h with a maximum of ~ 800 RLU (Fig. 6D orange line). In both strains, the addition of glucose pushed the peaks rightwards, presumably as the bacteria preferentially utilize the sugars. The large increase in expression from P_*atxA*_ is not concomitant with expression from P_*lef*_ indicating anthrose may modulate expression of *atxA* at the transcriptional and post-transcriptional level. In Fig. S5, pairwise absolute differences showed the similar effect of HIB + glucose and BHI on expression from P_*atxA*_ and P_*lef*_ , supporting the role of carbon metabolism in controlling toxin regulons.

### Exogenous anthrose and decoyinine have similar impacts on expression of important virulence promoters

**Figure 7:**
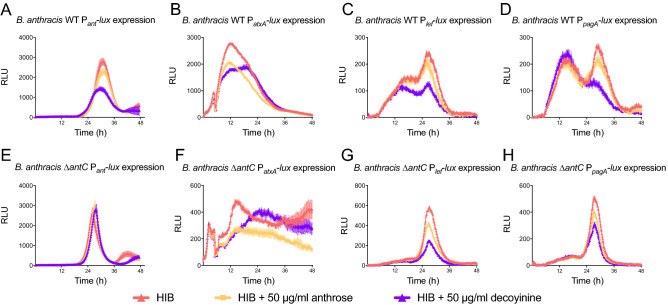
Anthrose and decoyinine have similar effects on expression profiles of toxin related genes. Luminescent expression patterns of (**A**) Sterne WT P_*ant*_-*lux* , (**B**) Sterne WT P_*atxA*_-*lux*, (**C**) Sterne WT P_*lef*_-*lux,* (**D**) Sterne WT P_*pagA*_-*lux,* (**E**) Sterne Δ*antC* P_*ant*_-*lux,* (**F**) Sterne Δ*antC* P_*atxA*_-*lux,* (**G**) Sterne Δ*antC* P_*lef*_-*lux* and (**H**) Sterne Δ*antC* P_*pagA*_-*lux* grown in HIB + Km 10 (red lines), HIB + Km10 + pure anthrose (orange lines), or HIB + Km10 + decoyinine (purple lines) show exogenous anthrose has similar impacts on gene expression as decoyinine in high protein, low sugar conditions; and mostly during stationary phase.

Anthrose as a highly unique sugar may play a role in intra, inter and extracellular signaling. It is well known *B. anthracis* responds strongly to nucleoside analogues as germinant molecules^31–33^. In addition, nucleoside-like molecules can trigger sporulation^34,35^. One such analogue is decoyinine, a nucleoside analogue produced by *Streptomyces* spp*.* It is a GMP synthetase inhibitor and *Streptomyces spp.* sporulation signal and triggers sporulation in *B. anthracis.* Decoyinine inhibits GMP synthesis thereby decreasing intracellular GTP levels leading to derepression of genes of the CodY repressive regulon^36^. The *atxA*, *lef*, and *pagA*, promoter fusions in the WT (Fig. 7A–D) and Δ*antC* Sterne (Fig. 7E–H) strains were grown in protein rich HIB medium (red curves) and with anthrose (orange lines) or decoyinine (purple lines). The addition of decoyinine decreased expression from the three constructs tested in both strains. Addition of the same amount of anthrose decreased expression similarly to decoyinine albeit an intermediate level. Pairwise distances between luminescent expression reporters show the similar effects of anthrose and decoyinine on expression profiles from *atxA*, *lef*, and *pagA* promoters in WT and Δ*antC* Sterne (Fig. S6).

**Figure 8:**
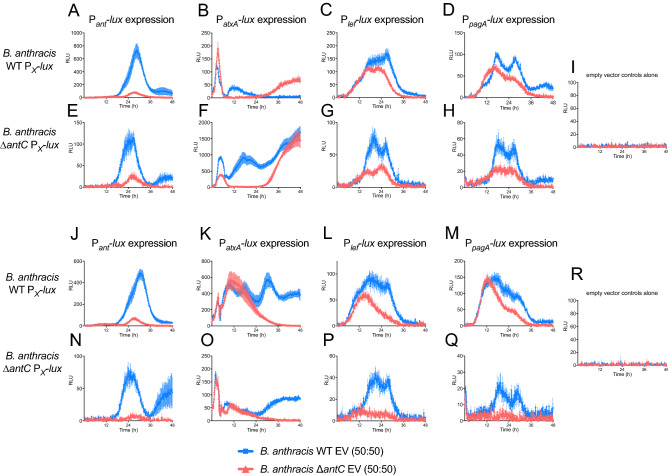
Co-culture with anthrose positive strains affects expression of virulence related and anthrose biosynthetic genes. Luminescent expression patterns of (**A**) Sterne WT P_*ant*_*-lux*, (**B**) Sterne WT P_*atxA*_*-lux*, (**C**) Sterne WT P_*lef*_-*lux,* (**D**) Sterne WT P_*pagA*_-*lux,* (**E**) Sterne Δ*antC* P_*ant*_*-lux*, (**F**) Sterne Δ*antC* P_*atxA*_*-lux*, (**G**) Sterne Δ*antC* P_*lef*_-*lux,* (**H**) Sterne Δ*antC* P_*pagA*_-*lux* when grown in BHI at a 50:50 mixture with Sterne WT EV strain (blue lines in each graph) or Sterne Δ*antC* EV strain (red lines in each graph). (**I**) Shows that pure cultures of either the Sterne WT EV strain or Sterne Δ*antC* EV strain are not luminescent. (**J–R**) are the same co-culture strains as (**A**–**I**) but were grown in HIB.

To see if expression of the anthrose operon and toxin genes can be modulated by natively relevant levels of external anthrose, WT Sterne and Δ*antC* Sterne strains containing the empty expression vectors were mixed in 50:50 ratios with the relevant luminescent fusion strains and grown in BHI broth (Fig. 8A–H) or HIB broth (Fig. 8J–R). In agreement with the studies where pure exogenous anthrose was added to cultures grown in BHI broth, co-culture with anthrose positive empty vector strains led to increased expression from the luminescent promoter fusions in both the WT and Δ*antC* Sterne backgrounds (blue lines), compared to co-cultures with the Δ*antC* Sterne empty vector strains (red lines). Interestingly, co-culture with the Δ*antC* empty vector strain reduced expression from the anthrose promoter (Fig. 8A, E, J and N; red lines compared to blue) regardless of media or anthrose status. Expression was consistently lower from the *lef* and *pagA* promoters when co-cultured with the Δ*antC* empty vector strain regardless of media or anthrose status of the reporter strain. In BHI, when the *atxA* fusions are grown in the empty vector anthrose cognate strain (like vs like) they express from P-_*atxA*_ as if they are grown in single BHI cultures (Fig. 8B and F). When either P-_*atxA*_ fusion is grown in HIB with the Δ*antC* mutant, expression from P-_*atxA*_ is similar to monocultures (Fig. 8K and O). As expected, empty vector strains alone did not generate any luminescent signals (Fig. 8I, R).

## Discussion

Our findings identified numerous *ant* ^*-*^* B. anthracis* isolates beyond the previously described WAG strains*.* What we first hypothesized as a genotype limited to West Africa encompasses isolates from around the world, including exported strains and those involved in human disease events. These *ant* ^*-*^ strains have been causing animal infections in the U.S. since at least 1960 (sheep isolate 2002013072). The aptly named heroin clade and the emerging ‘djembe’ clade are two major *ant*^*-*^ groups associated with dangerous high-profile exported anthrax events resulting in human cases. The presence of *ant*^*-*^ strains in the Ames clade (Han and FDAARGOS_694), South America, and WAG greatly expand observed *ant* ^*-*^ strains to many of the major A clades. We have also shown through analysis of the exosporium nap, that the absence of anthrose in the exosporium results in lower nap density and reduced molecular weight of the main exosporium protein BclA. Removal of anthrose changed the binding profile of the human AVA vaccine indicating a reduction in binding of vaccine specific antibodies to high molecular weight glycosylated BclA. This led us to probe the presence of spore specific antibody responses in immune serum. Antibodies to non-protein spore specific material were found in rabbit polyclonal serum to live spores, Sterne-vaccinated bison serum, and surprisingly AVA-vaccinated pooled human sera. The AVA vaccine is made from alum adsorbed vegetative cell filtrate and our data indicate components are present stimulating immune responses to protein and non-protein components of the *B. anthracis* spore. We have shown addition of exogenous anthrose causes transcriptional changes in vegetative bacteria as soon as 30 min post-exposure, linking a spore specific molecule to impacts on vegetative cell physiology. By 2 h post-exposure, sporulation specific proteins are upregulated. For example, *yfhD* is upregulated in the forespore in response to ethanol exposure or glucose deprivation in *Bacillus subtilis* ^37^. It is also the predicted location of a ncRNA in its 3’ end^38^. This gene experienced over 100-fold increase in expression 2 h after addition of pure anthrose. In *B. subtilis,* expression of *yfhD* is regulated by sigma-F, whose activity is derepressed during asymmetric septation of the forespore ^39^. Several hypothetical proteins on pXO1 experience significant levels of differential expression. These transcriptional changes could provide the driving force for mutation of the anthrose operon in vegetative cells. The compilation of data presented here provide more information towards the consequences of anthrose mutation.

We have shown that anthrose and decoyinine may act through similar pathways to control expression of toxins. Experiments showed that knocking out anthrose shifted induction from its own promoter to early stationary compared to late stationary in the WT. Addition of exogenous anthrose or decoyinine repressed expression from P_*ant*_ in the WT strain while having little effect on Δ*antC*; perhaps due to continued inability to produce anthrose. When anthrose was added to cultures containing BHI broth, which contains high levels of glucose, expression of the *pagA* promoter fusion was increased. This could be explained by the post-transcriptional control of *atxA* by the PTS sugar system and its link to growth phase dependent nutrient availability. CodY binds branched chain amino acids (BCAAs) and GTP, enhancing its affinity for its targets^40^. When BCAAs and/or GTP become limiting as during sporulation conditions in less complex media or treatment with anthrose or decoyinine in HIB, CodY is unable to bind to the promoter regions of the genes it regulates, leading to their derepression; this could include derepression of the thus far unidentified protease that has been hypothesized to post-translationally control AtxA levels. To summarize, the downstream effect of treatment with anthrose in rich media (BHI) is higher expression of *atxA* and higher levels of toxin expression due to the dominance of CodY-independent mechanisms of toxin regulation during logarithmic growth; such as regulation of *atxA* expression by AbrB^41^. In later phases of growth, CodY-dependent regulation of toxin regulation dominates and anthrose presence is coordinated with less active *atxA* and lower toxin expression. AbrB is a repressor of *atxA* and toxin expression. AbrB is normally inhibited by phosphorylated Spo0A (Spo0A ~ P) during logarithmic growth conditions allowing *atxA* and toxin expression^42^. Our data show that during logarithmic growth *atxA* expression and toxin expression is greatly repressed in the anthrose mutant compared to the wildtype Sterne. However, *atxA* expression levels were not coordinated with toxin expression. Low levels of *atxA* expression in the anthrose mutant suppress the initial peak of toxin expression found in the wildtype. The second peak in toxin expression is amplified in the anthrose mutant. Along those lines, addition of exogenous anthrose represses that second peak of toxin expression in both anthrose positive and anthrose negative Sterne, further supporting the role of anthrose in regulating toxin expression in the transition to stationary growth and beyond. Co-culture experiments showed that anthrose status of *B. anthracis*, the nutrient status of *B.* anthracis they grow with, and the nutrients they grow in can cause dissimilar shifts in expression of important virulence genes. These patterns need to be investigated further to assess the hierarchy of signaling through extracellular and intracellular pools of anthrose and how they affect virulence expression in vivo.

**Figure 9:**
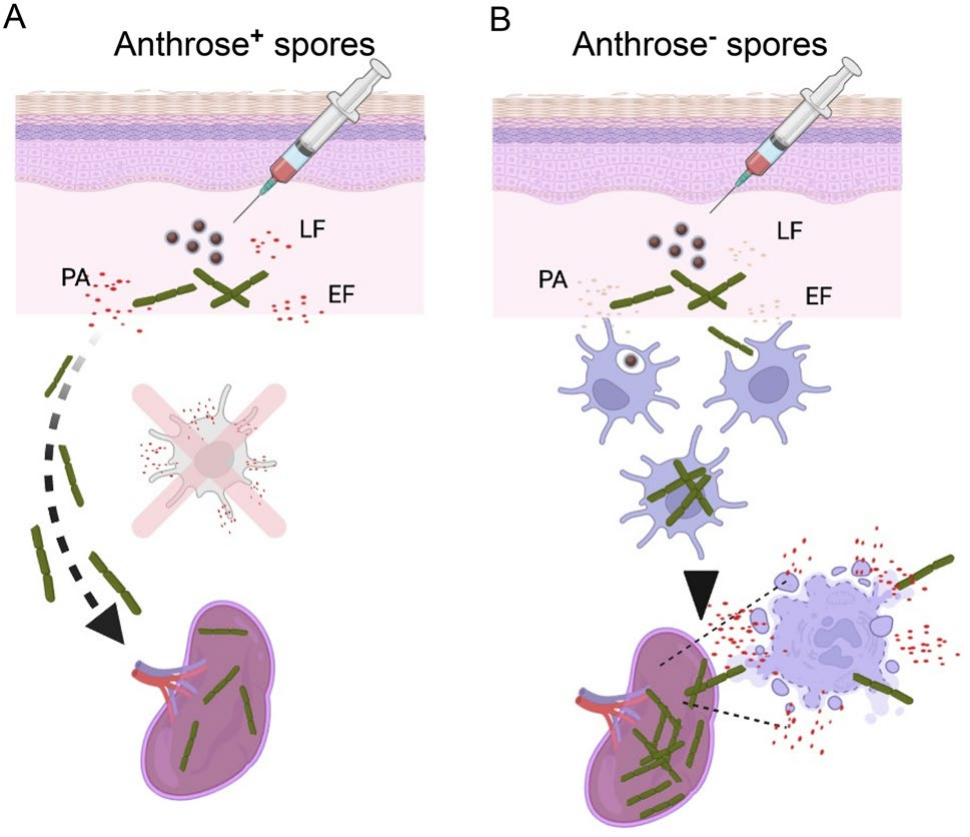
Model of anthrose status and dissemination in subcutaneous anthrax. (**A**) Anthrose positive spores germinate faster and elaborate more toxin during vegetative growth. Professional phagocytes at the site of infection are killed by the high levels of toxins. In the subcutaneous anthrax model, spread to secondary tissues primarily occurs after infection at local inoculation sites then through damaged lymphatics; as suggested in the jail-break hypothesis. (**B**) Anthrose negative spores germinate at a slower rate and secrete lower levels of toxin when they do germinate. Phagocytes survive the decreased levels of toxin, interacting at higher rates and phagocytizing anthrose negative spores more frequently than anthrose positive spores. Spores and vegetative cells are phagocytized, survive intracellularly and are trafficked to secondary tissues leading to higher levels of tissue dissemination; as in the trojan horse model. Higher levels of dissemination coincide with spikes in toxin secretion levels that accompany reduced mean time to death seen in anthrose negative spore infections. Created with BioRender.com.

We have generated a plausible model to help summarize our findings in the context of our previously published data on subcutaneous anthrax and anthrose negative spores (Fig. 9). Figure 9 shows how, in the course of subcutaneous infection, anthrose positive anthrax spores germinate and secrete toxin to enable local infections to spread systemically according to the jail-break hypothesis of dissemination (Fig. 9A). In contrast, anthrose negative spores do not germinate as fast and interact more efficiently with professional phagocytes at the site of inoculation, permitting phagocyte-assisted dissemination to secondary tissues as outlined in the trojan horse model of anthrax dissemination. Lower levels of toxin secretion by anthrose negative spores allow increased interaction between bacteria and phagocytes, a large spike in toxin secretion occurs when protein sources become limited, resulting in increased dissemination and shorter mean time to death in mice and *G. mellonella* as previously published. This model helps unify the in vitro and in vivo findings surrounding anthrose negative *B. anthracis*. In our previous work, we found a similar reduction in LD_50_ when mice were challenged with the anthrose negative mutant by the intranasal route^18^. Extrapolating from previously published data that show increased interaction of anthrose negative spores with macrophages and higher binding to the macrophage CD14 receptor, the trojan horse model of host-cell mediated dissemination could be favored. Anthrose negative spores could be cleared form the bronchoalveolar space faster than anthrose positive spores due to the increased interaction with phagocytes. Anthrax infection is a spectrum of dissemination where pathology is mediated by bacterial survival and toxin secretion in vivo. In this work, we focused on acapsular *B. anthracis* Sterne and the role of anthrose in toxin expression. Our future work will focus on testing this model by in vivo measurements of toxin secretion, pathogen spread, and cellular involvement and their impacts on pathogenesis by fully pathogenic encapsulated *B. anthracis*. The capsule is a major virulence factor whose regulation and induction can be controlled by *atxA* expression^43,44^ with both *atxA* and capsule expression linked to CO_2_ levels^45^. We observed effects of anthrose, both external and internal, on *atxA* expression while also showing there are likely other regulators that are involved. As we have teased apart the role of anthrose in toxin expression as part of this work, the next logical step is to understand if capsule expression in *B. anthracis* is affected and if virulence in animals can be impacted.

Sensing exogenous anthrose (in trans) has implications in pathogen ecology as well. Exogenous anthrose, whether it is attached to a spore surface or free floating, could provide signals that push vegetative cells towards sporulation, thus providing a means of spore-to-vegetative cell communication of sporulation-inducing conditions. Sugar residues with structures highly similar to anthrose are found in capsule produced under certain growth states by *Shewanella* spp*.* strain MR-4 and as glycosylations of the *Pseudomonas syringae* flagellum^46^. *Shewanella* spp. are found in anaerobic soil and water environments while *P. syringae* is a ubiquitous plant pathogen. The soil and plant environments are both places where *B. anthracis* would interact with these two bacteria under harsh environmental conditions. It would be interesting to assess whether the anthrose residues on these unrelated gram-negatives are sufficient to induce gene expression changes in *B. anthracis* that we observed here; providing another means for *B. anthracis* to sense unfavorable growth conditions.

## Methods

### Whole genome sequencing

gDNA was isolated from 1 ml of *B. anthracis* cultures grown in BHI broth overnight using the Dneasy UltraClean Microbial Kit (Qiagen: Germantown, MD, USA). DNA was filter sterilized using Corning Costar Spin-X 0.22 mm centrifugal filters. One tenth of the sample volume was inoculated into 3 ml of BHI broth with shaking. After 48 h, 100 ml of the sample was spread plate on BHI agar to verify sterility of the sample. Sterile samples were removed from high-containment and DNA was fragmented and prepared for sequencing using the NEBNext Ultra II FS DNA Library Prep kit (New England Biolabs: Ipswich, MA, USA) as recommended by the manufacturer. Samples were multiplexed using NEBNext Multiplex Oligos for Illumina (New England Biolabs; Ipswich, MA, USA). The 600-cycle MiSeq Reagent Kit v3 was used to sequence multiplexed samples on the MiSeq located in the Emerging Pathogens Institute at the University of Florida, Gainesville.

### Bioinformatic analysis

Blastn was used for initial identification of anthrose operon mutations. The HiPerGator high-performance research computing infrastructure at the University of Florida was used to analyze next-generation sequencing files. Fastq files were downloaded from the publicly accessible Sequence Read Archive (SRA) at the NCBI or the EMBL European Nucleotide Archive or from our own Illumina MiSeq runs. Raw reads were mapped to the *B. anthracis* Ames-ancestor using BWA-MEM^47^ and visualized using IGV^48^ for verification of anthrose mutations. De novo assembly of genomes was accomplished by trimming fastq files for quality using Trimmomatic^49^, assembled with SPAdes^50^, and polished with Pilon^51^. Genome assembly quality was verified using Quast^52^. Whole genome SNP analysis was performed using the PhaME package^53^ run on HiPerGator and trees were built using the RaxML option within PhaME^54^. Phylogenetic trees were visualized using iTOL^55^.

### Bacterial strains, growth conditions, and growth curves

*Escherichia coli* DH5α was used as a cloning strain and grown in LB broth or agar at 37 °C. The mobilizable strain RHO3 was grown in the presence of 200 μg/ml of DAP (Millipore Sigma: Burlington, MA, USA) for conjugating plasmids into wild-type *B. anthracis* carried out as previously described^56,57^. Counter selection was accomplished by omitting DAP from the selective medium. Kanamycin was used at 35 μg/ml in *E. coli* and 10 μg/ml in *B. anthracis* strains. *B. anthracis* Sterne 34F2 was obtained from Colorado Serum Company (USA) and grown with BHI broth or agar (Becton Dickinson: Franklin Lakes, NJ, USA) or heart infusion broth (HIB); (Research Products International: Mount Prospect, IL, USA) at 37 °C. Pure anthrose was purchased from Millipore Sigma (Burlington, MA, USA) and decoyinine was obtained from Abcam (Waltham, MA, USA). The *B. anthracis* Sterne 34F2 Δ*antC* mutant and complement were created as previously described^18^. Wild-type *B. anthracis* are from the Martin E. Hugh-Jones *Bacillus anthracis* Collection housed at the Emerging Pathogens Institute at the University of Florida. Bacteria were manipulated using BSL3 practices and procedures according to the BMBL in a CDC/USDA inspected and registered facility.

### Spore preparation and purification

Spores were prepared as previously described^18^ briefly *B. anthracis* strains were grown overnight in BHI broth, spread on Difco sporulation medium (DSM) agar plates, and incubated at 30 °C for 5 days. Spores were harvested into cold sterile water and purified through diatrizoic acid gradients. The pellets were resuspended in 85% ethanol for 1 h to kill any vegetative cell carry over then washed in cold water before enumeration and storage at 4 °C.

### High pressure freezing/freeze substitution, electron microscopy and image analysis

Freeze substitution and electron microscopy were performed as previously descried with minor modifications^58^.Spores were suspended in Trump’s Fixative (Electron Microscopy Sciences, Hatfield, PA) for 24 h and stored in 4 °C then pelleted at 10,000 rpm for 5 min (Fisher Scientific Micro-Centrifuge Model 59A). Cells were then washed in 0.1 M sodium cacodylate, pH 7.24 using a Pelco BioWave Pro laboratory microwave (Ted, Pella, Redding CA, USA). The spores were placed into a HPM100 3 mm, 200 μm deep type-A aluminum specimen carrier (Leica Microsystems, Vienna, Austria), prefilled with 1-hexadecene cryoprotectant. The spore-containing specimen carriers were covered with another type-A aluminum specimen carrier and high-pressure frozen using HPM 100 (Leica Microsystems, Vienna, Austria). The frozen samples were transferred into cryo-vials precooled in liquid nitrogen then filled 2% (w/v) osmium tetroxide and 0.1% uranyl acetate in anhydrous acetone. Freeze substitution was carried out in a freeze substitution unit (AFS2, Leica Microsystems, Vienna, Austria) at − 90 °C for 72 h, the temperature was stepped down with two further freeze substitution steps at − 50 °C for 24 h and − 10 °C for 24 h. After freeze substitution, the frozen samples were removed from the specimen carriers, washed in anhydrous acetone, and the temperature was raised to 4 °C. Resin infiltration steps were carried out in a laboratory microwave as described above. Dehydrated samples were infiltrated in graded acetone-Embed/Araldite epoxy resin with Z6040 embedding primer (Electron Microscopy Sciences, Hatfield, PA) at 30%, 50%, 70%, and 100% then cured at 70 °C. Ultra-thin sections (approximately 120 nm) were collected on 100 mesh Formvar/carbon-coated copper grids and counterstained with 2% aqueous uranyl acetate and Reynold’s lead citrate. Sections were examined with a FEI Tecnai G2 Spirit Twin TEM (FEI Corp., Hillsboro, OR) operated at 120 kV and digital images were acquired with a Gatan UltraScan 2 k × 2 k camera and Digital Micrograph software (Gatan Inc., Pleasanton, CA) at the University of Florida Interdisciplinary Center for Biotechnology Research electron microscopy core.

Electron micrographs were analyzed using Fiji^59^. Exosporium nap from 10 images of each strain was linearized using a 50-pixel line selection and the straighten function in Fiji. 3D-surface plot heat-maps were created by aligning the nap from 10 randomly selected spores using the 3D surface plot function. Histograms of the straightened images were created, and pixel density was determined.

### Western blots and ELISA

Samples for Western blots were collected and boiled in standard SDS-PAGE sample buffer for 10 min. The samples were run on pre-cast 4–15% SDS polyacrylamide gels (BioRad: Hercules, CA, USA) for 1 h at 120 V. Samples were electrotransferred onto methanol-soaked Immobilon P^SQ^ PVDF membranes (Millipore-Sigma: Burlington, MA, USA) using a semi-dry transfer at 17 V for 20 min. Blots were blocked with 5% skim milk in 1xPBS + 0.05% Tween 20 (PBST) for 1 h prior to washing 5 × in PBST for 3 min each. Primary antibodies were added at 1:1000 in blocking solution with gentle rocking for 1 h. After another five rounds of washing with PBST, detection antibodies were added at 1:1000 for colorimetric blots or 1:20,000 for chemiluminescent blots in blocking solution for 1 h then washed again as before. For colorimetric blots, 1-Step Ultra TMB Blotting solution (ThermoFisher Scientific: Waltham, MA, USA) was used as substrate while SuperSignal West Pico PLUS (ThermoFisher Scientific: Waltham, MA, USA) was the substrate for chemiluminescent blots. Both substrates were used according to the manufacturer’s recommendations. Blots were imaged on a BioRad Gel Doc XR + Gel Documentation system (Bio-Rad Laboratories; Hercules, CA, USA) in chemiluminescent mode with 60 s manual exposure. Goat anti-PA polyclonal antibody and recombinant PA were obtained from List Labs. Rabbit anti-BclA polyclonal antibody (NR-9578) was obtained from BEI resources. Mouse monoclonal antibodies LF-3H3 and LF-9A11 against LF (NR-12187 and NR-12188) and P2E3H4 against EF (NR-15473) were obtained from BEI Resources. Horseradish peroxidase (HRP)-conjugated secondary detection antibodies used were as follows; Goat anti-mouse IgG HRP (Invitrogen A16072), goat anti-human IgG HRP (Invitrogen 31,412), rabbit anti-goat IgG HRP (Sigma A8919), and goat anti-rabbit IgG HRP (Sigma A0545). Protective antigen was measured by ELISA with the Anthrax Protective Antigen 83 (PA83) Protein Quantitative ELISA (Alpha Diagnostics International; San Antonio, TX, USA; 800–100-P83) performed according to the manufacturer’s recommendations.

### RNA-seq experiments

*B. anthracis* Sterne strain 34F2 was grown overnight in BHI broth at 37 °C and standardized to an OD_600_ of 0.5 and divided into 6 tubes: triplicate for pure anthrose (Sigma) as treatment at a final concentration of 10 μg/ml, and triplicate for a mock of water added. Samples were collected in triplicate at 30 min and triplicate at 120 min after introduction of either liquid. Samples at each timepoint were immediately processed through the Zymo Direct-zol RNA Miniprep Plus (Zymo Resrearch; Irvine, CA, USA) kit including the optional bead beating steps as well as the on column DNAse treatment. Resulting RNA was quantified on a NanoDrop 2000 (Thermo Fisher Scientific; Waltham, MA, USA) and saved at –80 °C. To remove contaminating rRNAs ~ 1.6 μg of each sample was treated with Terminator™ 5′-Phosphate-Dependent Exonuclease (Lucigen; Middleton, WI, USA) and cleaned up using the Ambion MEGAclear Transcription Clean-Up Kit (Thermo Fisher Scientific; Waltham, MA, USA) while removing tRNAs and sRNAs. After an rRNA degradation check via a bleach RNA denaturing gel ^60^ sample quantity was standardized via NanoDrop 2000.

RNAseq libraries were prepared using the NEBNext® Ultra™ II RNA Library Prep Kit for Illumina® using the manufacturer’s recommendation. Resulting sample libraries were then assayed for correct sizing via a High Sensitivity DNA Analysis chip in a Bioanalyzer 2100 (Agilent; Santa Clara, CA, USA). The libraries were sequenced using an Illumina NovaSeq 6000. Resultant fastq files were submitted to the PATRIC RNA Seq Analysis pipeline^61,62^ using the Tuxedo suite^63^ to analyze transcripts between replicates and significance between treatments. Significant genes were visualized using GraphPad Prism. The raw and processed data from the experiment have been deposited in the Gene Expression Omnibus and are accessible through GEO Series accession number GSE220794 (https://www.ncbi.nlm.nih.gov/geo/query/acc.cgi?acc = GSE220794). String network analysis was used to identify clusters of gene regulation in these data sets^64^. Lines connecting genes are different evidence of interactions from the STING database which mines several bioinformatics resources.

### Construction of empty vector and complementation vectors

The pRepU-kan-AmCyan plasmid was created by PCR amplifying the backbone with phosphorylated oligos RepUKanCFPFOR (5′-Phos/tttcattggatcccccgggagatc-3′) and RepUKanCFPREV (5′-Phos/agtgagtcgacctcgacaaaaga-3′). The PCR product was digested with *Dpn*I to remove the plasmid template then self-ligated. The resulting plasmid was verified by *Bam*HI/*Kpn*I. The pRepU-kan-AmCyan-*antABCD*_*Sterne*_ plasmid was created by digesting pRP1099 ^65^ with *Bam*HI and *Sal*I and amplifying the *B. anthracis* Sterne *antABCD* operon by PCR with Q5 DNA polymerase and oligos AntAssemF2 (5′-acttcgttcttttgtcgaggCTTGTATTGTCCACTTATTTTATCCC-3′) and AntAssemR2 (5′-gatatcgagatctcccggggACATAATATCCCCTCATACACAC-3′). The NEBuilder HiFi DNA assembly kit was used to assemble the plasmid backbone and anthrose operon by Gibson assembly according to the manufacturer’s recommendations. Correct insertion was verified by double-digest of the insert with EcoRI and HindIII.

### Creation of luminescent expression vectors and luminescent expression analysis

The pRepU-kan-AmCyan plasmid was created as above. pRepU-kan-AmCyan-P_*pagA*_-*lux* was produced as previously described^66^. The gram-positive codon optimized and rearranged *luxABCDE* operon from pMV30*6-hsp-lux* was obtained from Addgene and was created as described in^67^. The *lef* promoter was amplified with Plef-Up_NotI (5′-TAT GCG GCC GCG CAA AAA ACA AAC TAA AT-3′) and Plef-Dn_EcoRI (5′-ATG AAT TCT CTC CTT TTT TAT AA GTG-3′) then digested with *Eco*RI and *Not*I then inserted into pMV306-P_*pagA*_-*lux*^66^ digested with the same enzymes to create pTs-repA-kan-P_*lef*_-*lux*. pRP1099 was digested with *Bam*HI and *Sal*I and was combined with the P_*lef*_-*lux* fragment amplified with NEBuildLEFLUXFWD (5′-TCT CGA CTT CGT TCT TTT GTC GAG GGC AAC GCG TGC G-3′) and NEBuildLEFLUXREV (5′-AAT TCG ATA TCG AGA TCT CCC GGG GTG ATC ACC GCG GCC ATG AT-3′) then assembled using the NEBuilder HiFi assembly kit. *Eco*RI and *Not*I digest confirmed a 250 bp band coinciding to the *lef* promoter and sequence verified by sanger sequencing with the sequencing oligo lux-seq (5′-CAA ACT CCG TGA AAT GAT GCT CC-3′). To create pRepU-kan-AmCyan-P_*sigF*_-*lux* oligos PspoIIAAsigF.FOR (5′-TCT CGA CTT CGT CGG CAA ATA TTT TGC CGC TTT TCT AT-3′) and PspoIIAAsigF.REV (5′-TTC CAA ATT TCA TAC GAT TTC CTC CTT ATG CTC AAA CTT TAC TAA TT-3′) amplified the *spoIIAA-spoIIAB-sigF* promoter and was assembled with the pRepU-kan-AmCyan-*lux* fragment amplified from pRepU-kan-AmCyan-P_*pagA*_-*lux* with oligos LuxPspoAAIIsigF.FOR (5′-GGA GGA AAT CGT ATG AAA TTT GGA AAC TTT TTG CTT ACA TAC CAA CCT CCC C-3′) and RepuKmPspoAA.REV (5′-AAA ATA TTT GCC GAC GAA GTC GAG ATC AGG GAA TGA GTT-3′). To create pRepU-kan-AmCyan-P_*ant*_-*lux* oligos Pant.FOR (5′-TCT CGA CTT CGT CCG AAG GAA TGT AAA GAT GAT TAA TAT GGT AGT AGA ATA ATT TAA AG-3′) and Pant.REV (5′-TTC CAA ATT TCA TAA AAA GTC CCC TTT TAA ATC CCT AAT TTT TCT-3′) amplified the *antABCDE* promoter and was assembled with the pRepU-kan-AmCyan-*lux* fragment amplified from pRepU-kan-AmCyan-P_*pagA*_-*lux* with oligos LuxPant.FOR (5′-AGG GGA CTT TTT ATG AAA TTT GGA AAC TTT TTG CTT ACA TAC CAA CCT CCC-3′) and RepUPant.REV (5′-TAC ATT CCT TCG GAC GAA GTC GAG ATC AGG GAA TG-3′). To create pRepU-kan-AmCyan-P_*atxA*_-*lux* oligos PatxA.FOR (5′-TCT CGA CTT CGT GTT CTA AAT CGT AAG GGG TTT TAT TAG TTA TAT TTC TTT TTT AGT TCA-3′) and PatxA.REV (5′-TTC CAA ATT TCA TGT CTA TAA TTG ATT CTC CTT TCC TGT TGT G-3′) amplified the *atxA* promoter and was assembled with the pRepU-kan-AmCyan-*lux* fragment amplified from pRepU-kan-AmCyan-P_*pagA*_-*lux* with oligos LuxPatxA.FOR (5′-TCA ATT ATA GAC ATG AAA TTT GGA AAC TTT TTG CTT ACA TAC CAA C-3′) pRepUKmPatxA.REV (5′-TAC GAT TTA GAA CAC GAA GTC GAG ATC AGG GAA TG-3′). Presence of each promoter was PCR confirmed and then sanger sequencing of each plasmid with oligo lux-seq (5′-CAA ACT CCG TGA AAT GAT GCT CC-3′) confirmed the expected sequence.

For luminescent growth curves, bacteria were grown in starter cultures of HIB over night with 10 μg/ml kanamycin where required. The OD_600_ was measured by diluting 1:10 in fresh media and back-calculating by the dilution factor. Cultures were normalized to an OD_600_ of 1 in fresh assay specific medium containing 10 μg/ml of kanamycin. Normalized cultures were used to inoculate the different media with 1:40 dilutions. 150 μl aliquots were grown in black 96-well cell repellent optical bottom plates (Greiner Bio-One; Monroe, NC, USA). Assays were performed in a Synergy Mx plate reader (BioTek; Winooski, VT, USA) at 37 °C with orbital shaking at 425 cpm. The optical density at 600 nm (OD600) and relative luminescent units (RLUs) were recorded every 10 min for 48 h. Each assay was carried out in biological triplicate and technical duplicate. Plate luminescence was imaged by spotting 20 μl of 1:40 diluted cultures onto HIB + Km10 agar plates and visualizing after 24 h growth at 37 °C as above for chemiluminescent western blots.

Co-culture experiments were performed by growing starter cultures as described above then mixing either the non-luminescent empty vector containing *B. anthracis* Sterne 34F2/pRepU-kan-AmCyan or the non-luminescent *B. anthracis* Sterne 34F2 Δ*antC*/pRepU-kan-AmCyan strains with the indicated luminescent reporter strains at a 50:50 ratio or alone in BHI + Km10 or HIB + Km10. Luminescent signals were measured as described in the other luminescent assays in this work.

### Analysis of luminescent expression plots

The difference between two time series can either be summarized as the set of differences between the values of the first and second series for each time point (which can either be positive or negative depending on which series value is bigger) or as the set of absolute differences for each time point (which can only be nonnegative). The number of elements in such sets corresponds to the number of compared time points between the two considered series.

The pairwise absolute differences for each time point were summarized visually to see how those differences were changing over time (Fig. S5). In addition to that, the differences were also presented as histograms, which illustrate whether one series tends to be larger than the other and how often. In Fig. S6 the pairwise differences with the corresponding sign for each time point were presented, where the values above zero indicate that the first compared series is larger than the second one and negative values indicate the opposite.

## Supplementary Information

Supplementary Information.

## Data Availability

All data from this work is available in the manuscript, its supplement, or online. The raw and processed data from the RNA-seq experiment have been deposited in the Gene Expression Omnibus and are accessible through GEO Series accession number GSE220794 (https://www.ncbi.nlm.nih.gov/geo/query/acc.cgi?acc=GSE220794).
