# Supplementary material for: Beyond the spore, the exosporium sugar anthrose impacts vegetative Bacillus anthracis gene regulation in cis and trans
Source: Sci Rep. 2023 Mar 28;13:5060. doi: 10.1038/s41598-023-32162-x (PMC10050317; doi:10.1038/s41598-023-32162-x)
Supplement: Supplementary file 1 — Supplementary Information. [file 41598_2023_32162_MOESM1_ESM.docx]

A

B

C

D

E

F

G

H

I

**Figure S1. TEM of purified *B. anthracis* Sterne spores.** *B. anthracis* Sterne spores were generated on Difco sporulation media and fixed overnight prior to high pressure freezing and imaging by TEM. A) The *B. anthracis* Sterne WT spores, B) Δ*antC*, and C) Δ*antC*/COMP spores were visualized and representative images of single spores of each are presented in D, E and F. Black boxes indicate areas zoomed for detail of the exosporium nap in G, H, and I. Brackets indicate the nap fibers as they extend from the exosporium surface.

A

E

D

C

B

**Figure S2. Reactivity of protein and non-protein components of vegetative cells and spores with immune sera.** *B. anthracis* Sterne samples (1, vegetative; 2, proteinase K treated vegetative cells; 3, purified spores; 4, proteinase K treated spores) were analyzed by SDS-PAGE. Total protein was visualized by Coomassie stain in A). B) The same samples blotted against rabbit polyclonal antibody to live Sterne spores. C) Western blot to detect protein and non-protein spore specific antibodies using pooled human AVA serum. D) Western blot of the same samples using *B. anthracis* Sterne vaccinated-bison serum. E) Western blot using naïve bison serum. L, broad range color protein standard; kDa, kilodalton.

**Figure S3. Light microscopy analysis of *B. anthracis* Sterne and *B. anthracis*** Δ***antC* Sterne.** Bacteria grown in HIB were visualized at 40x magnification at 24, 48, and 72 h post-inoculation.

Δ*antC/*

Δ*antC/*

Δ*antC/*

**Figure S4. Raw blots as source for band intensities of Fig 4.**

**Figure S5. Absolute difference plots for each time point and the corresponding histograms for**

**those differences of luminescent signal curves from *atxA* and *lef* promoters grown in different media.**

**Figure S6. Difference plots of *atxA, lef,* and *pagA* reporter strains for each time point grown with addition of anthrose or decoyinine.**

**Uncropped blots for Figure 2**

**Uncropped blots for Figure S2**

**Uncropped blots for Figure S4**
